# Prevalence and risk factors for HIV-1 infection in rural Kilimanjaro region of Tanzania: Implications for prevention and treatment

**DOI:** 10.1186/1471-2458-7-58

**Published:** 2007-04-19

**Authors:** Elia J Mmbaga, Akhtar Hussain, Germana H Leyna, Kagoma S Mnyika, Noel E Sam, Knut-Inge Klepp

**Affiliations:** 1 Department of General Practice and Community Medicine, University of Oslo, Oslo, Norway; 2 Department of Epidemiology and Biostatistics, Muhimbili University College, Dar es Salaam, Tanzania; 3 Department of Nutrition, University of Oslo, Oslo, Norway; 4 Department of Clinical Microbiology, Kilimanjaro Christian Medical Centre, Moshi, Tanzania

## Abstract

**Background:**

Variability in stages of the HIV-1 epidemic and hence HIV-1 prevalence exists in different areas in sub-Saharan Africa. The purpose of this study was to investigate the magnitude of HIV-1 infection and identify HIV-1 risk factors that may help to develop preventive strategies in rural Kilimanjaro, Tanzania.

**Methods:**

A cross-sectional study was conducted between March and May of 2005 involving all individuals aged between 15–44 years having an address in Oria Village. All eligible individuals were registered and invited to participate. Participants were interviewed regarding their demographic characteristics, sexual behaviors, and medical history. Following a pre-test counseling, participants were offered an HIV test.

**Results:**

Of the 2 093 eligible individuals, 1 528 (73.0%) participated. The overall age and sex adjusted HIV-1 prevalence was 5.6%. Women had 2.5 times higher prevalence (8.0% vs. 3.2%) as compared to men. The age group 25–44 years, marriage, separation and low education were associated with higher risk of HIV-1 infection for both sexes. HIV-1 infection was significantly associated with >2 sexual partners in the past 12 months (women: Adjusted odds ratio [AOR], 2.5 (95%CI: 1.3–4.7), and past 5 years, [(men: AOR, 2.2 (95%CI:1.2–5.6); women: AOR, 2.5 (95%CI: 1.4–4.0)], unprotected casual sex (men: AOR,1.8 95%CI: 1.2–5.8), bottled alcohol (Men: AOR, 5.9 (95%CI:1.7–20.1) and local brew (men: AOR, 3.7 (95%CI: 1.5–9.2). Other factors included treatment for genital ulcers and genital discharge in the past 1 month. Health-related complaints were more common among HIV-1 seropositive as compared to seronegative participants and predicted the presence of HIV-1 infection.

**Conclusion:**

HIV-1 infection was highly prevalent in this population. As compared to our previous findings, a shift of the epidemic from a younger to an older age group and from educated to uneducated individuals was observed. Women and married or separated individuals remained at higher risk of infection. To prevent further escalation of the HIV epidemic, efforts to scale up HIV prevention programmes addressing females, people with low education, lower age at marriage, alcohol consumption, condom use and multiple sexual partners for all age groups remains a top priority. Care and treatment are urgently needed for those infected in rural areas.

## Background

Diverse patterns of the spread of HIV-1 infection have been reported in sub-Saharan Africa. HIV-1 prevalence is reported to be declining or stabilizing in some populations in east Africa, while few areas still record an increasing epidemic [1-6]. Huge variation in transmission factors and preventive efforts in the presence of different stages of epidemic partly explain the differential spread of HIV-1 infection observed[1,3]. Studies in Africa and elsewhere have shown the rate of spread of HIV-1 infection to be higher in urban than in rural populations [1,3,7-12]. However, the inequalities in risk transmission factors such as poor access to or inadequate quality of treatment of sexually transmitted infections (STI), poor HIV/AIDS knowledge, poor condom supply and gender inequalities in rural areas makes these communities vulnerable for the acquisition of HIV-1 infections.

Expansion in surveillance systems covering rural and remote areas are now revealing some silent epidemics in rural areas[6,13]. The high and increasing prevalence of HIV-1 in some rural African populations indicates that without appropriate interventions, the impact of HIV/AIDS could be substantial in these communities[5,6,14-19]. Inequity in treatment access favoring urban and highly educated people in sub-Saharan countries is substantiated by the premise that the HIV burden is still low in rural communities[20,21]. Information on the magnitude of HIV in rural areas is a prerequisite to scaling up HIV prevention and treatment in these areas. Efforts to combining treatment with effective HIV-1 prevention remains a priority, calling for a continued monitoring of the magnitude of HIV-1 infections and the associated risk factors (socio-demographic and behavioural) fuelling the transmission.

In 1991 a survey of a village population in Kilimanjaro Tanzania found the age adjusted prevalence of HIV-1 infection to be 3.2% among individuals aged 15–44 years[22]. A follow-up study in 1993 showed the prevalence to have increased to 3.6% with an incidence of 4.3/1000 and 13.0/1000 Person-Years at Risk (PYAR) among men and women, respectively[22,23]. Additionally, married individuals had three times the risk of infection as compared to unmarried individuals[23]. Both studies reported multiple sexual partners to be the most likely risk factor for HIV infection in this community[22,23]. To establish how the HIV-1 epidemic and associated factors have evolved since early1990's in rural areas believed to be low transmission areas, we surveyed again the same village population. Therefore, the purpose of the present paper is two fold; firstly, to present current magnitude of HIV-1 infection in this rural population of Tanzania and secondly, to identify population specific HIV-1 risk factors with a view to develop community specific preventive and curative measures.

## Methods

### Study area

The study was conducted in Oria village in Kahe ward, Kilimanjaro, Tanzania. Details of this study area have been described elsewhere[10]. In brief; Oria is a rural village at the foot of Mount Kilimanjaro about 30 kilometres from Moshi town in Kilimanjaro region of Tanzania. The majority of the people engage in small scale rice and maize farming and some keep goats and/or cattle.

### Study design and population

A population based cross-sectional study was conducted between March and May of 2005. All individuals aged between 15–44 years (N = 2 093) and listed as having a permanent address in the village were eligible for participation.

### Data collection procedures

House-to-house registration of all eligible individuals was completed by the research team. The village had nine hamlets (small administrative units) and data collection was done from hamlet to hamlet. Dates to visit each hamlet for data collection were set by the research team and the hamlets leaders. At the set dates, the research team went from house-to-house. Participants received information regarding the study aims and procedures, and those who agreed to participate provided a written informed consent. A structured questionnaire was administered in a private place to ensure confidentiality. During the interviews information on the socio-demographic factors (age, sex, education, current occupation, marital status), risk behaviours (alcohol consumption, number of sexual partners in the past 12 months and 5 years, ever used condom, exchange of money/goods for sex) were collected. Health-related factors, namely history of ever treated for genital ulcer disease [GUD] or genital discharge syndrome [GDS] in the past 1 month, treatment for tuberculosis, herpes zoster in the past 5 years and having prolonged fever, diarrhoea or considerable weight loss in the past 1 month were also collected. For alcohol consumption, drinkers were considered as those who consume alcohol at least once per week. The interviews were followed by pre-test counselling and subsequent blood collection for HIV-1 testing. Appointments were made with each participant for post-test counselling within two weeks from the date of sample collection. Test results were taken to the village after two weeks of blood collection. Efforts were made to provide results to all who wanted to know their status as this would serve as an entry point for care and prevention. Individuals who indicated that they did not want to know their test results were also re-visited and re-counseled. All missed participants during recruitment were visited three times at home or at their nearby working place before being considered as non-respondents.

### Ethical considerations

Ethical approval for the study was provided by the Ethical Committee of the Ministry of Health in Tanzania and the Norwegian Committee for Medical Research Ethics in Norway. The village government of Oria granted permission for the study. All participants gave written informed consent for the interview and blood sampling separately. HIV-1 test results were issued to respondents in person after pre-test and post-test counselling. Further medical follow-up was offered for all HIV-1 positive respondents. All residents of the village had access to free treatment for common medical conditions from a research mobile clinic during the time of the survey.

### Laboratory methods

HIV-1 antibodies were detected using two independent enzyme-linked immunoassay (ELISA) systems (Voronostica Uniform II plus O; Organon, Boxtel, the Netherlands and Enzygnost Anti HIV 1/2 Plus; Dade Behring, South Africa). If the ELISA results were discordant or weakly concordant, Western Blot (Bio-Rad Laboratories Ltd, Dart ford, UK) was used for confirmation. All specimens were tested at the clinical laboratory of the Kilimanjaro Christian Medical Centre, Moshi, Tanzania.

### Statistical analysis

Descriptive statistics of the sample were obtained through frequencies and cross tabulations. The χ^2 ^test for differences in proportions was applied for categorical variables; continuous normally distributed variables were presented as means with their respective standard deviations (SD). To test for differences between means, the student t- test was employed. Overall and sex-specific HIV-1 prevalence's were calculated, and using the district population as standard population, these prevalence's were adjusted for age. Strength of association between HIV-1 infection and various risk factors was estimated by calculating the odds ratios (OR) with 95% confidence intervals (CI). Multiple logistic regression with HIV-1 seropositivity as a dependent variable was used to control for potential confounders. Adjusted odds ratios (AOR) with 95% CI were then presented. Due to differences in the rate of HIV-1 infection between women and men, all the analyses were stratified by sex. The Statistical Package for Social Sciences (SPSS) for windows version 13.0 (SPSS Inc., Chicago, IL, USA) was used for the data analysis.

## Results

A total of 914 (59.8%) women and 614 men participated in the study. The response proportions for the survey were 73.7 % (914/1 241) and 72.1% (614/852) for women and men, respectively. Sex composition (p = 0.524) and mean age [28.2 (8.4) versus 27.9 (8.6), p = 0.783] did not differ between participants and registered non-participants. The main reason for non-participation was short-term absence.

Of the 1 528 participants, 37 (2.4%) declined blood testing leaving 890 women and 601 men tested for HIV-1 antibodies. There was no difference in demographic characteristics or sexual behaviors related variables between those who accepted and those who declined blood testing. Table 1 depicts the distribution of sociodemographic characteristics of the study population. Age groups, marital status, education levels and religion affiliation composition did differ significantly by sex as presented in the table.

**Table 1:** Distribution of sociodemographic characteristics by sex among adults in Oria village, Kilimanjaro Tanzania

Variable	Category	Total	Women n (%)	Men n (%)	*p-value
Sex		1 528	914 (59.8)	614 (40.2)	
Age (years)	Mean (SD)	28.2 (8.4)	27.9 (8.6)	28.7 (8.8)	0.056
Age groups (years)					
	15–24	575	352 (38.5)	223 (36.3)	
	25–34	534	333 (36.4)	201 (32.7)	
	35–44	419	229 (25.1)	190 (30.9)	0.038
Marital status					
	Single	477	225 (24.6)	256 (41.7)	
	Married	899	577 (63.1)	322 (52.4)	
	Separated/divorced	148	112 (12.3)	36 (5.9)	<0.001
Education level					
	No education	191	125 (13.7)	66 (10.7)	
	Primary education	1196	716 (78.6)	480 (78.2)	
	Secondary +	138	70 (7.7)	68 (11.1)	0.027
Occupation					
	Farmers	1327	799 (87.4)	528 (86.0)	
	Employed	49	25 (2.7)	24 (3.7)	
	Business	152	90 (9.9)	62 (10.1)	0.429
Religion affiliation					
	Catholic	392	226 (24.9)	166 (27.1)	
	Protestant	458	257 (28.3)	201 (32.8)	
	Muslim	672	426 (46.9)	246 (40.1)	0.031

* P value from Chi-square test for difference in proportions, SD: Standard deviation.

The overall crude prevalence of HIV-1 infection was 6.4% (96/1 491). When adjusted for age and sex, the adjusted prevalence was 5.6%. The adjusted prevalence was 2.5 times higher among women as compared to men (8.0% for women and 3.2% for women). The prevalence of HIV-1 infection increased significantly with increase in age groups for both women and men (p_trend _< 0.001) (Table 2). The odds of HIV infection was almost three times among women aged above 24 years as compared with those aged 24 years and below. The corresponding odds of HIV infection among men was five times higher among the older groups (>24 years) (Table 2). The study subjects who reported to be married or separated had higher prevalence as compared to those reported to be single. Increase in educational attainment was associated with less risk of infection as compared to those with no education. However, this association was only significant among men.

**Table 2:** Associations between demographic characteristics and HIV-1 infections by sex among adults in Oria village, Kilimanjaro Tanzania

Variable	Category	Women (n = 890)			Men (n = 601)		
			
		No	HIV+	Multivariate*	No	HIV+	Multivariate*
Age groups(years)^¶^	15–24	340	3.5	1	219	1.0	1
	25–34	327	10.4	2.5 (1.2–5.2)	197	5.1	5.1 (2.4–9.1)
	35–44	223	13.0	2.8 (1.3–6.2)	185	5.9	5.3 (2.5–7.4)
							
Marital status	Single	202	3.5	1	266	1.8	1
	Married	577	8.5	1.9 (1.1–3.5)	299	5.7	2.4 (1.5–11.1)
	Separated	111	18.9	3.3 (1.2–9.0)	36	5.9	2.6 (1.3–17.8)
							
Polygamous marriage^‡^	No	523	7.6	1	277	5.3	1
	Yes	54	9.3	1.1 (0.5–2.6)	22	9.1	1.6 (0.3–7.7)
							
Education level	No educ	122	13.4	1	59	6.8	1
	Primary educ	698	7.6	0.5 (0.3–1.9)	471	3.2	0.4 (0.1–0.9)
	Secondary+	70	8.6	0.7 (0.3–2.5)	71	2.8	0.3 (0.1–0.6)
							
Occupation	Farmers	805	8.2	1	545	3.5	1
	Employed	24	4.2	0.5 (0.1–4.5)	24	0.0	0.0 (0.0-0.0)
	Business	61	13.1	1.7 (0.7–3.8)	32	6.3	2.8 (0.5–13.9)
							
Religion	Catholic	223	7.7	1	163	6.2	1
	Protestant	254	9.9	1.4 (0.7–2.8)	197	3.0	0.4 (0.1–1.2)
	Muslim	413	8.0	1.1 (0.6–2.1)	241	2.1	0.3 (0.1–0.8)

*Adjusted for age (continues variable), marital status, education level and religion, ¶: p trends across age-groups < 0.001 for both sexes, ‡: From those who report being married.

In this study population, 25.1% of women and 5.6% of men reported to have married or cohabited at less than 17 years of age. The risk of HIV-1 infection among these individuals was higher compared to that of those who married or cohabited at age 17 years and above (Table 3). A substantial proportion of women (11.9% and 21.8%) and men (26.5% and 46.1%) reported to have ≥ 2 sexual partners during the past 12 months and 5 years, respectively. Having >2 sexual partners both in the past 12 months and 5 years was associated with two and a half times higher odds of HIV-1 infection among women. For men, significant increased risk was only seen among those reporting >2 sexual partners in the past 5 years. Self-reports on ever used a condom indicated that 31.8% of women and 52.1% of men had ever used. Among those reported to have had casual sex, 31.6% of women and 47.4% of men reported to have used condom at last casual sex. However, men who did not use condom during casual sex had 1.8 (95% CI: 1.2–5.8) times increased odds of infection compared to condom users. Regarding the consumption of local brew, 12.2% of women and 16.3% of men (p = 0.022) reported using local brew at least once per week. Bottled alcohol was consumed by 2.6% and 4.4% of women and men, respectively. Alcohol consumption both bottled and local was significantly associated with higher risk of HIV-1 infection among men. No corresponding association was observed among women.

**Table 3:** Associations between reported risk behaviours and HIV-1 infections by sex among adults in Oria village, Kilimanjaro Tanzania

Variable	Category	Women (n = 890)			Men (n = 601)		
			
		No	HIV+	Multivariate*	No	HIV+	Multivariate*
Age first married/cohabited^‡^	≥ 17	431	7.5	1	258	5.0	1
	<17	146	12.2	1.5 (1.1–2.9)	41	12.5	2.9 (0.5–14.)
							
Age at sexual debut	≥ 17	434	8.8	1	246	3.7	1
	<17	352	10.1	1.2 (0.7–2.0)	282	4.3	1.6 (0.6–4.1)
							
Number of partners past 12 months^#^	0–1	692	8.3	1	388	3.1	1
	≥ 2	94	17.4	2.5 (1.3–4.7)	140	5.0	1.7 (0.6–4.7)
							
Number of partners past 5 years^#^	0–1	614	7.6	1	285	2.5	1
	≥ 2	172	15.1	2.4 (1.4–4.0)	243	5.3	2.2 (1.2–5.6)
							
Ever used condom^#^	Yes	250	8.8	1	275	3.2	1
	No	536	10.4	1.2 (0.7–2.1)	253	4.7	1.4 (0.6–3.5)
							
Used condom during last casual sex^§^	Yes	103	8.7	1	137	3.2	1
	No	220	15.0	1.9 (0.7–4.3)	152	6.5	1.8 (1.2–5.8)
							
Exchanged money/goods during last sex^#^	No	713	8.9	1	450	3.8	1
	Yes	73	13.7	1.9 (0.8–4.2)	78	4.0	1.0 (0.3–3.6)
							
Used bottled alcohol	No	864	8.4	1	581	2.9	1
	Yes	26	8.7	1.0 (0.2–4.5)	20	20.0	5.9 (1.7–20.1)
							
Used local brew	No	779	7.7	1	502	2.2	1
	Yes	111	13.6	1.4 (0.7–2.6)	99	10.1	3.7 (1.5–9.2)

*Adjusted for age (continues variable), marital status, education level and religion, ‡: From those reported being married, §: From those reported to have casual sex, #: From those reported to have had sex.

Past month treatment for GUD was significantly associated with HIV-1 infection among women but not among men (Table 4). On the other hand, treatment for GDS in the past month was significantly associated with HIV-1 infection among men, but not among women. Reported health complaints were more common among the HIV-1 seropositive as compared to the HIV-1 seronegative individuals. As compared to HIV-1 seronegative individuals, a significant proportion of the HIV-1 seropositive individuals (5.2% versus 1.1%, p = 0.013) reported to have been treated for pulmonary tuberculosis. Similar findings was observed for herpes zoster (3.1% versus 0.6%, p = 0.008), prolonged fever (11.5% versus 3.7%, p < 0.001), prolonged diarrhea (3.1% versus 0.8%, p = 0.022) and considerable weight loss (14.6% versus 4.2%, p < 0.001). Associations between these variables are presented in Table 4 and demonstrate that most of these complaints were significantly associated with HIV-1 infection.

**Table 4:** Associations between HIV-1 infection and reported health related factors by sex among adults in Oria village, Kilimanjaro Tanzania

Variable	Category	Women (n = 890)			Men (n = 601)		
			
		No	HIV+	Multivariate*	No	HIV+	Multivariate*
Treated for GUD past 1 month	No	878	8.1	1	590	3.6	1
	Yes	12	36.4	6.4 (1.7–24.1)	11	9.0	2.1 (0.4–39.2)
							
Treated for GDS past 1 month	No	856	8.4	1	585	3.2	1
	Yes	34	9.1	1.0 (0.2–2.8)	16	12.5	4.2 (1.0–26.0)
							
Treated for TB past 5 years	No	876	8.1	1	593	3.5	1
	Yes	14	30.8	5.2 (1.5–18.2)	7	14.0	3.1 (0.0–31.4)
							
Treated for herpes zoster past 5 years	No	881	8.3	1	597	3.5	1
	Yes	9	25.0	3.8 (0.7–21.0)	5	20.0	4.3 (0.0–40.4)
							
Prolonged fever past 1 month	No	847	7.7	1	581	3.4	1
	Yes	43	23.8	3.6 (1.6–7.9)	20	5.0	1.4 (0.2–1.5)
							
Prolonged diarrhoea past 1 month	No	882	8.2	1	595	6.8	1
	Yes	8	37.5	7.2 (1.5–33.5)	6	16.6	2.3 (0.0–20.2)
							
Considerable weight loss past 1 month	No	847	7.6	1	572	3.1	1
	Yes	43	25.6	4.0 (1.8–8.6)	29	10.3	3.5 (1.0–12.7)

*Adjusted for age (continues variable), marital status, education level and religion; GUD, Genital ulcer disease; GDS, genital discharge syndrome; TB, Tuberculosis.

## Discussion

The results from this study show that the prevalence of HIV-1 infection was high in this rural community. Compared to men, women had two and half times higher HIV-1 prevalence. Above the age of 24 years, marriage, separation and lack of education are among the demographic factors associated with HIV-1 infection for both women and men. Risk behaviors fueling the spread of HIV-1 epidemic included age at first marriage/cohabiting, multiple sexual partners and treatment for GUD in the past 1 month preceding the survey among women. For men, multiple sexual partners, unprotected casual sex, alcohol consumption and treatment for GDS in the past 1 month preceding the survey were important risk factors associated with HIV-1 transmission. Most health-related complaints were predictors of HIV-1 infection.

The previous study from this rural population in 1991 showed a prevalence of HIV-1 infection among individuals aged 15–44 years of 3.2% (4.4% for women and 1.8% for men)[10,22]. This prevalence was consistent with prevalence data reported from other studies involving rural populations conducted at the same time elsewhere in Tanzania [9,11,17]. The high incidence (8.9/1000PYAR) reported in the 1993 follow-up study indicates a growing epidemic. Therefore, the current finding showing much a higher prevalence in this rural adult population (6.4% unadjusted)) suggests that the rate of HIV-1 infection may be increasing in this community. This is in line with other published studies in Tanzania[6,15,17,24]. It has been reported that despite the observed decrease in HIV-1 prevalence in some populations in Africa, variability in prevalence exists between and within countries[1,3]. This is because the HIV-1 epidemic is at different stages in different places and that variation in socio-cultural and economic factors as well as the degree of preventive efforts has affected the magnitude and direction of the epidemic in different ways. Thus, these findings confirm that the magnitude of HIV-1 infection is high in some rural populations in Tanzania, warranting coordinated care and prevention efforts.

In this population, women, individuals above the age of 24 years, marriage/separation and lack of education were associated with increased risk of HIV-1 infection. In the earlier studies in this population, young females, married individuals and those with higher education were at higher risk of infection as compared to other groups [23]. In this study 15 years later, women and married individuals were still at risk. However, there has been a shift of risk from younger to older age groups (>24 years) and from educated to uneducated individuals. This finding of a shift in the epidemic to older individuals is consistent with what has recently been reported in Zimbabwe and South Africa [25,26]. An increased focus on prevention among young people, might have contributed to a prevention gap and consequently, the epidemic has shifted to the older generation[3,26,27]. The decreased HIV-1 prevalence rate among educated individuals is in line with current findings in Zambia and Uganda [4,28,29]. This has been described as a result of the role of education as a "social vaccine" against HIV infection by contributing to delayed sexual debut and adoption of other safer sexual behaviors [4,28,30].

Bars and local clubs are places where people meet. It is recognized that alcohol may lead in to decreased sexual inhibition, impaired judgment and multiple sexual partners [31,32]. Alcohol consumption was associated with HIV-1 infection among men but not among women in this population. In a rural setting, men are more likely to consume alcohol than women. The majority of bar workers in Tanzania are women and are considered to be sex workers[33]. This means that men who consume alcohol come into contact with sex workers hence at higher risk of HIV-1 infection as observed in this study. Therefore, alcohol consumption might have contributed to the observed increase in HIV-1 infection in this population. The level of knowledge on HIV/AIDS transmission and prevention was high in this population[23]. This could be due to repeated HIV/AIDS health education and condom distribution delivered to all participants during previous surveys [10,23]. The high knowledge could have caused desirability bias, resulting in observed high rates of reported condom use in this population as compared to the national demographic survey[34]. As a result of this bias, people practising high-risk sexual behaviors are more likely to report condom use affecting the desired relationship between condom use and HIV-1 infection as observed in this study and others[34]. There is sufficient evidence that HIV infection does yield to determined intervention measures [26,35]. Prevention strategies work better when they are culturally and socially sensitive and tailored according to the intended community and addressing local HIV transmission factors.

The findings in this study have important public health implications. The prevalence of HIV-1 infection is high in rural Kilimanjaro Tanzania. Older women were at the highest risk of infection. We have identified important factors that could be targeted for HIV-1 prevention. Designing and intensifying prevention efforts with a particular focus on older uneducated married or separated women could prove beneficial. Promoting the reduction of multiple sexual partners and alcohol consumption with inclusion of local brew clubs in prevention may lead to desirable consequences of curbing newer infections. In this study we also found that a substantial proportion of those infected had health-related complaints pertaining to AIDS. It is reported that by august 2006, 2 million people were living with HIV infection in Tanzania, 100 689 were enrolled in treatment but only 48 013 were receiving antiretroviral therapy (ART) from both public and private sectors including ART projects [21]. Almost all of these people were from urban areas where public and private facilities are located. This underpins the existing inequality in ART provision efforts between urban and rural communities in Africa. Results from this study and that of others [6,14,17,19,22], highlight the need to strengthen ART provision strategies in rural communities. This is more important because treatment scale-up increases opportunities for undertaking effective prevention.

In this village survey, we achieved a response proportion of 73.0%. A response proportion above 70.0% is acceptable for population based studies. Although the demographic characteristics of those who did not participate did not differ with those who participated in this study, there is a potential for bias due to the fact that variables of interest were not measured from those who were missed. Experience from recent demography and health surveys in Africa indicates that, although the predicted HIV prevalence seems to be higher among no-responders, their effect on the overall national estimates were insignificant [36]. Therefore, these results may be representing the HIV-1 situation in this village population. Most of respondents in this study accepted a blood test for HIV-1 antibodies. Repeated HIV/AIDS education and counseling offered in our previous studies may have increased peoples awareness including importance of knowing their HIV status. This could partly explain the high response rate on blood testing observed in this study. Due to the inherent weakness of the cross-sectional study design, we could only describe the independent association between various factors and HIV-1 infection. This means that we can not establish causal- effect relationships between HIV-1 and those factors. However, these factors have also been described in stronger designs to be associated with HIV infection [5,17,23,24] securing their importance in this population.

## Conclusion

We have shown the prevalence of HIV-1 infection to be high in rural areas of Tanzania. As compared to our findings in the early 1990's [10,23] the epidemic seems to have shifted from younger to older and educated to uneducated populations. Women and married individuals were still at risk in this rural community. Lower age at marriage/cohabiting, alcohol consumption, multiple sexual partners, unprotected casual sex, and past 1 month treatment for GUD/GDS were important risk factors for HIV-1 transmission. A substantial number of those infected present with AIDS-related symptoms and may be in need of ART. As a comprehensive response, prevention efforts should be intensified to target and addresses identified transmission factors and include people of all age. Local brew bars should be targeted to capture those at risk of HIV infection. Concurrent attempts to expand access to treatment and care for those infected in rural areas are needed as this will increase opportunity for prevention.

## Competing interests

The authors declare that they have no competing interests.

## Authors' contributions

EJM conceived the study, coordinated data collection, carried out the statistical analyses and drafted the manuscript. AH participated in the study design, data interpretation and reviewed the manuscript. GHL involved in study design, data collection and critically reviewed the manuscript. KSM participated in data acquisition and reviewed the manuscript. NES supervised data collection, testing of samples and reviewed the manuscript. KIK coordinated the design and protocol, data analysis, interpretation of results and reviewed the manuscript. All authors read and approved the final draft of the paper.

## Pre-publication history

The pre-publication history for this paper can be accessed here:


